# 6-(1*H*-Tetra­zol-5-yl)-1*H*-indole monohydrate

**DOI:** 10.1107/S1600536811003990

**Published:** 2011-02-23

**Authors:** Yu-Hua Ge, Pei Han, Ping Wei, Ping-Kai Ou-yang

**Affiliations:** a College of Life Science and Pharmaceutical Engineering, Nanjing University of Technology, Nanjing, People’s Republic of China; b Department of Chemistry and Chemical Engineering, Southeast Universiy, Nanjing 211189, People’s Republic of China

## Abstract

In the title compound, C_9_H_7_N_5_·H_2_O, the tetra­zole ring forms a dihedral angle of 1.82 (1)° with the mean plane of the indole fragment. In the crystal, mol­ecules are linked by inter­molecular O—H⋯N, N—H⋯O and N—H⋯N hydrogen bonds into a two-dimensional network parallel to (100). Addtional stabilization is provide by weak π–π inter­actions with a centroid–centroid distance of 3.698 (2) Å.

## Related literature

For the synthesis and pharmacological activity of compounds containing indole and tetra­zole groups, see: Itoh *et al.* (1995 ▶); Semenov (2002 ▶). For the synthesis of 6-cyano­indole, a starting material for the title compound, see: Frederick (1949 ▶).
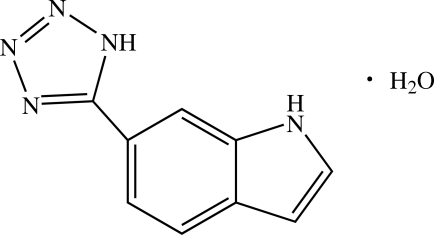


## Experimental

### 

#### Crystal data

C_9_H_7_N_5_·H_2_O
*M* *_r_* = 203.21
Monoclinic, 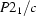

*a* = 17.175 (3) Å
*b* = 4.0653 (8) Å
*c* = 14.421 (3) Å
β = 107.59 (3)°
*V* = 959.8 (3) Å^3^
*Z* = 4
Mo *K*α radiation
μ = 0.10 mm^−1^
*T* = 293 K
0.20 × 0.05 × 0.05 mm

#### Data collection

Rigaku Mercury2 diffractometer
Absorption correction: multi-scan (*CrystalClear*; Rigaku, 2005 ▶) *T*_min_ = 0.737, *T*_max_ = 1.000
7430 measured reflections
1683 independent reflections
945 reflections with *I* > 2σ(*I*)
*R*_int_ = 0.120

#### Refinement

*R*[*F* ^2^ > 2σ(*F* ^2^)] = 0.066
*wR*(*F* ^2^) = 0.131
*S* = 1.01
1683 reflections
144 parameters
H atoms treated by a mixture of independent and constrained refinement
Δρ_max_ = 0.15 e Å^−3^
Δρ_min_ = −0.19 e Å^−3^

### 

Data collection: *CrystalClear* (Rigaku, 2005 ▶); cell refinement: *CrystalClear*; data reduction: *CrystalClear*; program(s) used to solve structure: *SHELXS97* (Sheldrick, 2008 ▶); program(s) used to refine structure: *SHELXL97* (Sheldrick, 2008 ▶); molecular graphics: *SHELXTL* (Sheldrick, 2008 ▶) and *DIAMOND* (Brandenburg, 2006 ▶); software used to prepare material for publication: *SHELXTL*.

## Supplementary Material

Crystal structure: contains datablocks I, global. DOI: 10.1107/S1600536811003990/lh5195sup1.cif

Structure factors: contains datablocks I. DOI: 10.1107/S1600536811003990/lh5195Isup2.hkl

Additional supplementary materials:  crystallographic information; 3D view; checkCIF report

## Figures and Tables

**Table 1:** Hydrogen-bond geometry (Å, °)

*D*—H⋯*A*	*D*—H	H⋯*A*	*D*⋯*A*	*D*—H⋯*A*
O1—H1*A*⋯N2^i^	0.90 (4)	2.07 (4)	2.957 (4)	169 (4)
O1—H1*B*⋯N3^ii^	0.76 (5)	2.17 (5)	2.927 (5)	172 (5)
N4—H4*N*⋯O1	0.86	1.87	2.715 (4)	169
N5—H5*N*⋯N1^iii^	0.86	2.17	3.019 (4)	171

Symmetry codes: (i) 
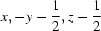
; (ii) 
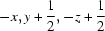
; (iii) 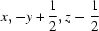.

**Fig. 1.**
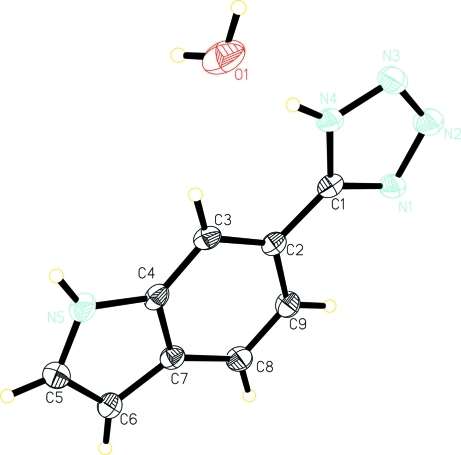
The molecular structure of the title compound with the atom numbering scheme. Displacement ellipsoids are drawn at the 30% probability level. H atoms are presented as a small spheres of arbitrary radius.

**Fig. 2.**
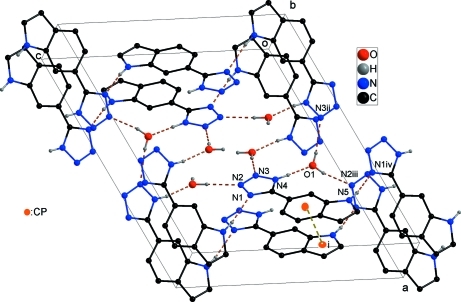
Part of the crystal structure with hydrogen bonds and π–π interactions shown as dashed lines. Only H atoms involed in hydrogen bonds are shown. CP denotes a ring centroid. [Symmetry codes: (i) x, y-1,z; (ii) -x+1, y+1/2, -z+1/2; (iii)x, -y+1/2, z-1/2; (iv) x, -y+3/2, z-1/2]

## supplementary crystallographic information

### Comment

In recent decades, there have been some reports on the compounds which are synthesized by the combination of the tetrazole and indole rings (Itoh *et al.*,1995) and property studies reveals that these compounds always perform unique pharmacological activities (Semenov *et al.*, 2002). In order to obtain such compounds, we have attempted to synthesize the indole compounds with tetrazole as a substituent. Herein, we report the crystal structure of the title compound (I). The molecular structure of (1) is shown in Fig. 1.

The indole unit is essentially planar, with a mean deviation of 0.007 (8)Å from the least-squares plane defined by the nine constituent atoms. The dihedral angle formed by the indole plane and the tetrazole ring is 1.82 (1)°. The crystal packing (Fig. 2) is stabilized by intermolecular O-H···N, N—H···O and N—H···N hydrogen bonds (Table 1). Further stabilization is provided by aromatic π–π interactions with a Cg1···Cg2(x, 1+y, z) distance of 3.698 (2) Å (Cg1 and Cg2 are the centroids of the N5/C4-C7 and C2-C4/C7-C9 rings, respectively).

### Experimental

All chemicals used (reagent grade) were commercially available. 6-Cyanoindole was synthesized following the methods described by Frederick (1949). To the stirring DMF solution of NaN_3_ and triethylamine, 6-cyanoindole was added. Then the mixture was heated to 120, about 1 h later, the solution was cooled to room temperature, and DMF was distilled in a vacuum. With some follow-up treatment, the crude product was recrystallized in methanol solution and seven days later, yellow prism crystal was obtained.

### Refinement

H atoms bound to C and N atoms were placed in calculated positions and refined using a riding model, with C—H = 0.94Å and *U*_iso_(H) =1.2*U*_eq_(C) or N—H = 0.86Å and *U*_iso_(H) =1.5*U*_eq_(N) . The H atoms of the water molecule were located in a difference map and refined freely.

### Crystal data

**Table:**

C_9_H_7_N_5_·H_2_O	*F*(000) = 424
*M**_r_* = 203.21	*D*_x_ = 1.406 Mg m^−^^3^
Monoclinic, *P*2_1_/*c*	Mo *K*α radiation, λ = 0.71073 Å
Hall symbol: -P 2ybc	Cell parameters from 2795 reflections
*a* = 17.175 (3) Å	θ = 3.1–27.5°
*b* = 4.0653 (8) Å	µ = 0.10 mm^−^^1^
*c* = 14.421 (3) Å	*T* = 293 K
β = 107.59 (3)°	Needle, colorless
*V* = 959.8 (3) Å^3^	0.20 × 0.05 × 0.05 mm
*Z* = 4	

### Data collection

**Table:**

Rigaku Mercury2 diffractometer	1683 independent reflections
Radiation source: fine-focus sealed tube	945 reflections with *I* > 2σ(*I*)
graphite	*R*_int_ = 0.120
Detector resolution: 13.6612 pixels mm^-1^	θ_max_ = 25.0°, θ_min_ = 3.2°
CCD_Profile_fitting scans	*h* = −20→20
Absorption correction: multi-scan (*CrystalClear*; Rigaku, 2005)	*k* = −4→4
*T*_min_ = 0.737, *T*_max_ = 1.000	*l* = −17→17
7430 measured reflections	

### Refinement

**Table:**

Refinement on *F*^2^	Primary atom site location: structure-invariant direct methods
Least-squares matrix: full	Secondary atom site location: difference Fourier map
*R*[*F*^2^ > 2σ(*F*^2^)] = 0.066	Hydrogen site location: inferred from neighbouring sites
*wR*(*F*^2^) = 0.131	H atoms treated by a mixture of independent and constrained refinement
*S* = 1.01	*w* = 1/[σ^2^(*F*_o_^2^) + (0.0398*P*)^2^] where *P* = (*F*_o_^2^ + 2*F*_c_^2^)/3
1683 reflections	(Δ/σ)_max_ < 0.001
144 parameters	Δρ_max_ = 0.15 e Å^−^^3^
0 restraints	Δρ_min_ = −0.19 e Å^−^^3^

### Special details

**Table:**

Geometry. All esds (except the esd in the dihedral angle between two l.s. planes) are estimated using the full covariance matrix. The cell esds are taken into account individually in the estimation of esds in distances, angles and torsion angles; correlations between esds in cell parameters are only used when they are defined by crystal symmetry. An approximate (isotropic) treatment of cell esds is used for estimating esds involving l.s. planes.
Refinement. Refinement of F^2^ against ALL reflections. The weighted R-factor wR and goodness of fit S are based on F^2^, conventional R-factors R are based on F, with F set to zero for negative F^2^. The threshold expression of F^2^ > 2sigma(F^2^) is used only for calculating R-factors(gt) etc. and is not relevant to the choice of reflections for refinement. R-factors based on F^2^ are statistically about twice as large as those based on F, and R- factors based on ALL data will be even larger.

### Fractional atomic coordinates and isotropic or equivalent isotropic displacement parameters (Å^2^)

**Table:**

	*x*	*y*	*z*	*U*_iso_*/*U*_eq_	
O1	0.0699 (2)	−0.0517 (10)	0.1761 (2)	0.0720 (10)	
H1A	0.088 (2)	−0.161 (10)	0.132 (3)	0.095 (17)*	
H1B	0.033 (3)	0.048 (11)	0.147 (3)	0.10 (2)*	
N1	0.19161 (16)	−0.0016 (7)	0.5257 (2)	0.0453 (8)	
N2	0.12138 (17)	−0.1708 (7)	0.5143 (2)	0.0518 (9)	
N3	0.08061 (16)	−0.1971 (7)	0.4227 (2)	0.0509 (9)	
N4	0.12440 (15)	−0.0389 (7)	0.37351 (19)	0.0415 (8)	
H4N	0.1108	−0.0189	0.3113	0.062*	
N5	0.32387 (16)	0.6304 (7)	0.21458 (19)	0.0442 (8)	
H5N	0.2898	0.6026	0.1576	0.066*	
C1	0.19266 (19)	0.0829 (8)	0.4367 (2)	0.0349 (8)	
C2	0.25600 (18)	0.2697 (8)	0.4122 (2)	0.0325 (8)	
C3	0.25007 (18)	0.3476 (8)	0.3176 (2)	0.0349 (8)	
H3	0.2048	0.2852	0.2666	0.042*	
C4	0.31364 (19)	0.5219 (8)	0.3008 (2)	0.0346 (8)	
C5	0.3976 (2)	0.7902 (8)	0.2348 (2)	0.0436 (9)	
H5	0.4185	0.8834	0.1884	0.052*	
C6	0.4357 (2)	0.7929 (8)	0.3320 (2)	0.0394 (9)	
H6	0.4862	0.8864	0.3636	0.047*	
C7	0.38315 (18)	0.6239 (8)	0.3762 (2)	0.0339 (8)	
C8	0.38770 (19)	0.5422 (8)	0.4718 (2)	0.0403 (9)	
H8	0.4327	0.6052	0.5231	0.048*	
C9	0.32503 (18)	0.3679 (8)	0.4894 (2)	0.0382 (9)	
H9	0.3280	0.3136	0.5530	0.046*	

### Atomic displacement parameters (Å^2^)

**Table:**

	*U*^11^	*U*^22^	*U*^33^	*U*^12^	*U*^13^	*U*^23^
O1	0.057 (2)	0.112 (3)	0.0391 (17)	0.0266 (19)	0.0019 (15)	−0.0135 (18)
N1	0.0348 (18)	0.058 (2)	0.0385 (18)	−0.0073 (16)	0.0049 (14)	0.0043 (16)
N2	0.0431 (19)	0.069 (2)	0.040 (2)	−0.0068 (17)	0.0080 (16)	0.0037 (17)
N3	0.0424 (19)	0.064 (2)	0.045 (2)	−0.0102 (16)	0.0113 (16)	0.0047 (17)
N4	0.0327 (16)	0.056 (2)	0.0329 (16)	−0.0036 (15)	0.0047 (14)	0.0050 (15)
N5	0.0454 (18)	0.057 (2)	0.0279 (16)	0.0038 (16)	0.0071 (13)	0.0024 (14)
C1	0.032 (2)	0.037 (2)	0.032 (2)	0.0057 (16)	0.0037 (16)	−0.0009 (16)
C2	0.0322 (19)	0.034 (2)	0.0292 (19)	0.0031 (16)	0.0062 (15)	−0.0008 (15)
C3	0.0297 (18)	0.043 (2)	0.030 (2)	0.0060 (17)	0.0047 (15)	−0.0045 (16)
C4	0.038 (2)	0.040 (2)	0.0256 (19)	0.0101 (18)	0.0093 (16)	0.0014 (16)
C5	0.038 (2)	0.046 (2)	0.048 (2)	0.0017 (19)	0.0154 (18)	0.0049 (19)
C6	0.0371 (19)	0.045 (2)	0.034 (2)	−0.0005 (18)	0.0075 (17)	0.0021 (17)
C7	0.034 (2)	0.037 (2)	0.0288 (19)	0.0045 (16)	0.0072 (16)	−0.0011 (16)
C8	0.035 (2)	0.051 (2)	0.029 (2)	−0.0065 (17)	0.0010 (16)	−0.0045 (17)
C9	0.041 (2)	0.047 (2)	0.0240 (19)	−0.0011 (18)	0.0052 (16)	−0.0010 (16)

### Geometric parameters (Å, °)

**Table:**

O1—H1A	0.90 (4)	C2—C9	1.417 (4)
O1—H1B	0.77 (4)	C3—C4	1.383 (4)
N1—C1	1.333 (4)	C3—H3	0.9300
N1—N2	1.355 (3)	C4—C7	1.413 (4)
N2—N3	1.298 (3)	C5—C6	1.355 (4)
N3—N4	1.344 (3)	C5—H5	0.9300
N4—C1	1.344 (4)	C6—C7	1.429 (4)
N4—H4N	0.8600	C6—H6	0.9300
N5—C5	1.374 (4)	C7—C8	1.397 (4)
N5—C4	1.380 (4)	C8—C9	1.375 (4)
N5—H5N	0.8600	C8—H8	0.9300
C1—C2	1.456 (4)	C9—H9	0.9300
C2—C3	1.373 (4)		
			
H1A—O1—H1B	106 (4)	N5—C4—C3	130.1 (3)
C1—N1—N2	106.5 (3)	N5—C4—C7	107.0 (3)
N3—N2—N1	110.6 (3)	C3—C4—C7	122.9 (3)
N2—N3—N4	106.4 (2)	C6—C5—N5	110.5 (3)
C1—N4—N3	109.3 (3)	C6—C5—H5	124.8
C1—N4—H4N	125.3	N5—C5—H5	124.8
N3—N4—H4N	125.3	C5—C6—C7	106.6 (3)
C5—N5—C4	108.7 (3)	C5—C6—H6	126.7
C5—N5—H5N	125.7	C7—C6—H6	126.7
C4—N5—H5N	125.7	C8—C7—C4	118.2 (3)
N1—C1—N4	107.2 (3)	C8—C7—C6	134.5 (3)
N1—C1—C2	126.7 (3)	C4—C7—C6	107.3 (3)
N4—C1—C2	126.2 (3)	C9—C8—C7	119.4 (3)
C3—C2—C9	120.6 (3)	C9—C8—H8	120.3
C3—C2—C1	121.7 (3)	C7—C8—H8	120.3
C9—C2—C1	117.7 (3)	C8—C9—C2	121.0 (3)
C2—C3—C4	117.8 (3)	C8—C9—H9	119.5
C2—C3—H3	121.1	C2—C9—H9	119.5
C4—C3—H3	121.1		
			
C1—N1—N2—N3	1.0 (4)	C2—C3—C4—N5	179.7 (3)
N1—N2—N3—N4	−0.8 (4)	C2—C3—C4—C7	−0.6 (4)
N2—N3—N4—C1	0.2 (4)	C4—N5—C5—C6	−0.7 (4)
N2—N1—C1—N4	−0.9 (4)	N5—C5—C6—C7	0.1 (4)
N2—N1—C1—C2	179.6 (3)	N5—C4—C7—C8	−179.8 (3)
N3—N4—C1—N1	0.4 (4)	C3—C4—C7—C8	0.5 (5)
N3—N4—C1—C2	179.9 (3)	N5—C4—C7—C6	−0.8 (3)
N1—C1—C2—C3	−178.9 (3)	C3—C4—C7—C6	179.4 (3)
N4—C1—C2—C3	1.6 (5)	C5—C6—C7—C8	179.2 (3)
N1—C1—C2—C9	1.5 (5)	C5—C6—C7—C4	0.4 (3)
N4—C1—C2—C9	−177.9 (3)	C4—C7—C8—C9	−0.2 (5)
C9—C2—C3—C4	0.4 (5)	C6—C7—C8—C9	−178.8 (3)
C1—C2—C3—C4	−179.1 (3)	C7—C8—C9—C2	0.1 (5)
C5—N5—C4—C3	−179.4 (3)	C3—C2—C9—C8	−0.2 (5)
C5—N5—C4—C7	0.9 (3)	C1—C2—C9—C8	179.4 (3)

### Hydrogen-bond geometry (Å, °)

**Table:**

*D*—H···*A*	*D*—H	H···*A*	*D*···*A*	*D*—H···*A*
O1—H1A···N2^i^	0.90 (4)	2.07 (4)	2.957 (4)	169 (4)
O1—H1B···N3^ii^	0.76 (5)	2.17 (5)	2.927 (5)	172 (5)
N4—H4N···O1	0.86	1.87	2.715 (4)	169
N5—H5N···N1^iii^	0.86	2.17	3.019 (4)	171

Symmetry codes: (i) *x*, −*y*−1/2, *z*−1/2; (ii) −*x*, *y*+1/2, −*z*+1/2; (iii) *x*, −*y*+1/2, *z*−1/2.
